# Needs assessment for home-based care and the strengthening of social support networks: the role of community care workers in rural South Africa

**DOI:** 10.3402/gha.v8.29265

**Published:** 2015-12-18

**Authors:** Mosa Moshabela, Ilona Sips, Francoise Barten

**Affiliations:** 1 Discipline of Rural Health, School of Nursing and Public Health, University of KwaZulu-Natal, Durban, South Africa; 2 International Centre for Health Systems Research and Education, Department for Health Evidence, Radboud University, Nijmegen, Nijmegen, The Netherlands

**Keywords:** community health worker, primary caregiver, patient-centred care, home-based care, social support networks, quality of care, South Africa

## Abstract

**Background:**

Community care workers (CCWs) in rural South Africa provide medical, personal, household, educational, and social care services to their clients. However, little understanding exists on how provision of services is approached within a household, taking into account available social support networks.

**Objective:**

The aim of this study was to generate an understanding of the processes that underpin the provision of care by CCWs in rural households and their engagement with clients, primary caregivers (PCGs), and other members of the social support network.

**Design:**

We analysed in-depth interviews conducted in a triad of participants involved in a home-based care (HBC) encounter – 32 clients, 32 PCGs, and 17 CCWs. For each triad, a purposefully selected CCW was linked with a purposefully selected client and the corresponding PCG using maximum variation sampling. Three coders used an inductive content analysis method to describe participants’ references to the nuances of processes followed by CCWs in servicing HBC clients. Written informed consent was obtained from all participants.

**Findings:**

The results suggest that, by intuition and prior knowledge, CCWs treated each household uniquely, depending on the clients’ care needs, cooperation, availability of a social network, and the reliability and resilience of the social support system for the client. Four distinct processes took place in rural households: needs assessment for care, rationing of care, appraisal of care, and reinforcement of a social support system. However, there was no particular order or sequence established for these processes, and caregivers followed no prescribed or shared standards.

**Conclusions:**

CCWs bring a basket of services to a household, but engage in a constant, dynamic, and cyclical process of weighing needs against services provided. The service package is uniquely crafted and tailored for each household, depending on the absorptive capacity of the social support network available to the client, and preferences of the clients remain central to the process of negotiating care.

Due to the significant increase in the burden of diseases in South Africa (1), families and communities are increasingly taking responsibility for providing care, as the health system struggles to cope with high demand (2). Consequently, up to 90% of illness care may be provided at home, thereby increasing capacity to provide health care for chronically-ill patients (3). The largest share of care is provided by family members, nearly always women, who serve as primary caregivers (PCGs) and mostly by taking on household chores (4, 5). Families are assisted in care provision by community care workers (CCWs), who are recruited from within and around the local community by home-based care (HBC) organisations and trained to provide basic services as volunteer caregivers to people in their home (5). CCWs are increasingly acknowledged to form a crucial part of the human resource pool for health care, particularly in resource-limited settings (6). Several studies have demonstrated the importance of the role of CCWs in sharing the burden of care with the formal health care system, as well as contributing to improvement in certain health outcomes (7).

In developing countries, the role of CCWs is mainly located in the space between the community and the formal health care systems, largely acting as facilitators of health care services in the community (8). Although many recognise their contribution in terms of bridging the social, cultural, and economic distances often found between the community and the formal health care system, the details of how CCWs manage care provision are often not well understood and are generally declared to constitute levels in quality of service delivery that are substandard (6). There is also a general assumption that the work of CCWs is standardised and structured, as suggested in the literature by the notion of ‘packages of care’ (3). However, care provision patterns by CCWs may differ and may depend on the gender, employment, and living arrangements of both the client and PCG (9). Evidence about task division between CCWs and PCGs is limited in resource-poor settings, a knowledge gap that should be addressed. Research regarding care division is often performed in the context of developed countries, and particularly with regards to care for the elderly (10). The existing evidence is primarily focused on who is involved in these tasks and describes the activities in which they are engaged (9). However, little is known with regards to systematic approaches to such task-sharing arrangements among those involved in caring for home clients. Without coordinated task-sharing efforts, the burden of care may become overwhelming for both the CCW and the members of the social support network providing the needed HBC (11, 12).

Research in social support networks for HBC should take into account the specific roles occupied by various actors in the social networks and support systems of the clients, as well as the poverty of resources prevalent in developing countries and rural contexts. In this study, we refer to a social support network as a network of people, including family members, neighbours, or friends, who support and assist the client with different care tasks in the household, defined by Whittaker and Garbarino (13). Approaches to the division of labour and task-sharing have not been adequately studied in the context of social support networks in rural and low-income settings. There is a need to explore in detail processes that underlie the sharing of tasks and responsibilities between the CCW and the social support network of the client, which was the main aim of this study. More specifically, we sought to answer the following questions: 1) How do CCWs approach need for care in the home for their clients? 2) How is the package of services for a given client decided upon? 3) How are care tasks divided, shared, and provided in the context of the social support network for the client? A better understanding of the nature of interactions between the CCW and the expanded social support network of carers, particularly the division and sharing of labour in HBC, can help inform further planning of these services in the backdrop of ongoing health reforms and shrinkage of resources.

## Methods

### Study design

We conducted in-depth, explorative, qualitative interviews based on triads of care (CCW–client–caregiver), inclusive of CCWs linked to their current clients, who were in turn linked to their main PCG. The qualitative study design was chosen based on the open-ended nature of research questions, necessitating an exploratory in-depth approach due to the need to understand the context-specific phenomenon of care provision within varying social support networks. Given the complex and dynamic nature of these social support networks, triangulation of data provided by three key members of the social support network was used in this study, specifically to increase the richness of data descriptions collected within a support network, to provide a basis for data comparison between participants in the same network, and to increase the trustworthiness of study findings within each triad of care (14). According to Flick, triangulation of data involves data drawn from different sources, at different times, in different places, or from different people (15), the latter most relevant to this study. Seale explains how data triangulation can be used to increase the scope, depth, and consistency of qualitative research findings, and not only as a form of data validation (16). The study formed part of a larger research project designed to investigate quality of care in the home in rural South Africa.

### Study setting

The study was conducted in the subdistrict of Bushbuckridge, Mpumalanga Province, in the Lowveld region of South Africa, located near the Kruger National Park. According to the 2011 national census, in this area, there were 542,000 inhabitants. The densely populated area was considered one of the 22 most poverty-stricken areas in South Africa, and a nodal area for development. There were three hospitals, two health centres, and in 2010, there were 37 known non-profit organisations (NPOs) providing HBC services through CCWs in the region (17).

### Study sampling

We conducted a situational analysis of all NPOs in the area, for which further details are described by Moshabela et al., with respect to the context, formation, and operations of these NPOs (17). From this work, we purposefully selected nine organisations based on diversity criteria intended to ensure a wide-ranging representation of NPO characteristics, further described by Sips et al. (8). Characteristics that were considered included the size of NPOs with regards to ratio of carers to clients, access to funding sources, care and service packages, geographic location, provision of stipends to carers, official registration with government, and relationships with local communities. We recruited two CCWs from the NPO employee databases, based on diversity criteria that included age, gender, education, training, experience, stipend, patient load, and village, and they were stratified by NPOs. We then collected lists of current patients for each CCW, from which we purposefully sampled two of their clients, again using diversity criteria that included age, gender, education, diagnosis, and type of care provided. For each client, we identified their main PCG. In so doing, we completed CCW–client–PCG triads. In the end, 17 CCWs and 32 clients with the corresponding number of PCGs were included. Therefore, a stratified purposeful sampling method with the aim of achieving maximum variation was used in this study (18).

### Data collection

Data collection took place between April and December 2010. Three teams comprised of two trained fieldworkers were each allocated three NPOs to conduct face-to-face interviews with participants using a topic guide. Topic guides used a handful of open-ended questions related to care services, and further questions were generated through probing. Questions for clients included the following: What has your experience been with your illness? What types of services or assistance do you need? What types of services are your CCW and PCG providing to you? What are your feelings about the state of services being provided to you? Questions for the PCG were very similar, but adapted to the caregiver role. For the CCW, questions included background information to their role, training, supervision, and organisational support, and questions were tailored to the specified client and their PCG with regards to the nature of needs, care services, support systems, and relationships. Interviews took place at a location preferred and chosen by the participants and lasted between 20 and 60 min. Most CCWs were interviewed privately in their NPOs, but a small minority were interviewed in their own homes. CCWs led the research team to homes of the selected clients, where an interview was conducted privately with clients, after they had provided consent and decided on their preferred arrangement for the interview. Similarly, PCGs were also interviewed in their homes, and most were interviewed on the same day as the clients, but return visits were often made with a scheduled appointment to interview the balance of some PCGs and clients. Participants in each triad were interviewed privately and independently using the language of their preference, Sepedi, Xitsonga, or Siswati. Within the research team, one member acted as an interviewer, and the other as participant observer, who captured field notes. Interviews were voice-recorded, translated, and transcribed into English for analysis. Quality checks were separately done by a senior qualitative interviewer for each interview transcript by comparing voice-recordings with transcripts. Additional interviews and member-checking were conducted for a subset of participants, eight CCWs, eight clients, and seven PCGs, whose interviews were incomplete or needed further clarification. Validation through member-checking was done at this stage to increase credibility of the qualitative data, in addition to the triangulation methods used (16).

### Data analysis

The data were managed through QSR International's NVivo 9 software, and transcripts were initially coded openly by three researchers independently using inductive conventional content analysis method. Codes related to approaches tocare by CCWs were generated from each transcript by each researcher, and these initial codes were compared and categorised. These peer-auditing procedures were also used to ensure the dependability, confirmability, and authenticity components of trustworthiness in qualitative data analysis (16). The constant comparison approach was used to elicit relations of codes and categories within participant groups, between participant groups, within participant triads and networks, and between triads and networks. Therefore, the next step in the analysis was to compare codes and categories between the same groups of participants, that is, CCW comparisons across the data. Codes and categories were revised and compared between participants within the same triad and social support network. This multilevel form of constant comparison using open coding, axial coding, and triangulation was also described by Boeije (19). Codes were further categorised and grouped into themes and subthemes and compared between triads and social support networks. Transcripts were read again to identify supporting and contradictory quotes using directed content analysis (16). Data are presented in this study according to themes and subthemes and taking into account constant comparison within and between triads of care and social support networks. Table 1 demonstrates the process of analysis moving from extract statements to codes, subthemes, and themes.

**Table 1:** Examples of transitions from textual extracts to codes, subthemes, and themes

Extracts from textual data	Codes	Subthemes	Themes
*Most places I just evaluate* [what care is needed] *and see how I can help them*.	Evaluate and decide on needs	Defining needs	Assessing needs for care
*I accepted it and they asked me what they should help me with for that day*.	Express willingness to accept care	Negotiating care	
*I do not cook for her because she does not want me to cook for her*.	Unique preferences of clients	Respecting boundaries	
*Each day I see seven clients and it depends on the conditions of the clients*.	Adjusting schedule based on priority	Tailoring of needs	Rationing of care
*She cooks food for the patient and asks about her pills. We would never ask her to do anything like sweep the yard*.	Subjectively ranking what is important	Value judgements	
*If they had an iron, I would iron their clothes. They have to be clean like I am*.	Using the ‘if’ and the ‘self’ as reference	Intuitive subjective assessment	
*There is a young lady* [CCW] *who comes to check on me but she does nothing*.	CCW seen to do ‘nothing’ when help is available	Critique of tailored care	Appraisal of care
*They always do any of these above-mentioned things if my daughter has not done them*.	CCW adjusting support as needed	Monitoring and tailoring of care	
*When I go to greet them, she* [PCG] *does tell me that my coming is enough because* [client] *would never allow me to touch any of her things*.	CCW support appreciated even when ‘nothing’ done	Appreciation of tailored care	
*My wife used to bathe me after work, my mother gave me food during the day, and my sister was the one taking me to hospital*.	Division of labour by all within a strong social support network	Functional social support networks	Reinforcing the social support network
*Last year she* [PCG] *was involved in a car accident and her hand got broken*.	Inability of PCG to help within the social support network due to chronic injury	Weakened social support network	
*She* [CCW] *helps with fetching water and believe me, if it was not for her, we do not know how things would be in this house*.	CCW playing an important role in closing gaps in care provision within a compromised social support network	Failing social support networks	
*Or we simply ask him which of his neighbours he gets along very well with*.	Identifying neighbour as a possible reinforcer of the social support network	Expanding social support networks	

CCW, community care worker; PCG, primary caregiver.

### Ethics review

The study was approved by the Human Research Ethics Committee at the University of Witwatersrand, Johannesburg, South Africa, under study protocol clearance number M090232. Further permission to conduct the study was given by the Mpumalanga Health Research and Ethics Committee. Permission was also requested from each NPO and participant approached as part of the parent study. Participation in this study was voluntary, and all participants were made aware that withdrawal from the study would by no means affect their care services. Verbal and written informed consent was obtained from all participants, for both the interview and voice recording. The consent process was documented in the field notes, and participants were provided with information sheets including contact details. Personal identifying information has been removed from all data used for analysis and presentation in this manuscript.

## Results

### Sociodemographic characteristics of participants

Almost all CCWs were female except for two males. The majority were aged between 25 and 44 years, with only four above 60 years of age. All CCWs had received some form of schooling, but only one had attended post-secondary school education. Most CCWs had received some form of training in TB or HIV care, including counselling and testing. However, only five CCWs received formal training for HBC. Approximately half of CCWs received some form of remuneration, while the other half worked voluntarily with no source of income. Most clients were female (20/32), and their ages ranged from 25 to 82 years. The majority were enrolled in HBC because of either TB or HIV/AIDS-related illnesses (21/32) and the rest had diabetes, hypertension, stroke, asthma, or some form of disability (11/32). Most participants lived with their family members (29/32), and only three lived on their own. Nearly all PCGs lived with their clients in the same household (27/32). Most PCGs were closely related to the clients, and largely in the form of a life partner (15/32), offspring (12/32), or parent (5/32). Grandchildren, siblings, cousins, and neighbours also acted as PCGs, albeit very seldom.

The results of this study are presented in four distinct but interconnected and overlapping themes, all of which relate back to the main study question on approaches to care in a household used by CCWs in a rural setting: assessment of needs for care, rationing of care, appraisal of care, and reinforcement of a social support network system. We elaborate on each theme below.

### Assessing needs for care

Needs assessment was the first and essential step in the care process. The evaluation of needs emerged as a subjective exercise, which the CCW carried out in two ways. First, the CCW would observe, examine, and interpret the physical and emotional condition of the client and the household and identify care needs based on this observation. Second, the client and family members were asked by the CCW to verbally express their struggles and needs.

#### Defining needs for care

Based on the observation of the CCW and the needs expressed by the client and family, the CCW would prioritise or rank the needs likely to guide care provision. The decision-making process was conducted either explicitly or implicitly, since some sort of decision had to be made as to what the clients and family needed most, captured in the quote below by a CCW:

When I arrive in the family for the first time I just evaluate what they need most. I also give them a choice to tell me their problems. (…) Most places I just evaluate and see how I can help them. (CCW 17 about Client 37, male, mobility impairment)

#### Negotiating care provision

The client of the CCW above confirmed that the approach used by the CCW when first visiting the household was that of negotiation and centred on the client's perceived needs and preference for assistance.

They [CCWs] arrived here telling me that they were working for a home-based care organisation and they asked me if they could come and assist me on daily basis; I agreed. Therefore they told me how they were going to provide me assistance. I accepted it and they asked me what they should help me with for that day. They just swept my room and I told them that when they visit me in the future I would like them to bathe me. They appreciated it and started to visit me on a daily basis. (Client 37 from CCW 17, male, mobility impairment)

#### Respecting boundaries of care

On the one hand, willingness to help and to provide services had to be shown by the CCWs, and the clients had to demonstrate their expressed willingness to cooperate and to accept HBC services. On the other hand, CCWs had to identify unique client preferences and respect boundaries drawn by such clients and/or their family members regarding care services to be provided, as shown hereunder.

I visit her to clean her yard, wash her clothes, and wash dishes. I do not cook for her because she does not want me to cook for her. (…) I do ask her daughter in law [PCG 26] to clean the house or something if I happen not to show up. But she [Client 26C] does not allow even her [PCG 26] to cook. She cooks for herself. (CCW 11, of Client 26, female, high blood pressure)

When the client (26C) was interviewed, the claim made by the CCW that the client refused cooking services was confirmed. Cooking was a service the client did not want from the CCW, and this choice was expressed as stated below.

I refused that she [CCW 11] should cook for me. My son said that I should get someone to cook for me, I refused as well. (Client 26, female, high blood pressure)

The sense of autonomy and self-determination created for clients in HBC appears central to the continuous relationship between clients and their CCWs. Therefore, the balance of power in the negotiation of care within a household context appears to be largely in favour of the clients. In assessing and deciding on the package of care needed, CCWs may insist that certain types of care be provided should failure to do so be perceived to be potentially detrimental to the health or well-being of the client. However, CCWs do not seem to wield absolute power or authority to compel clients or family to accept care beyond the bounds of negotiation and consensus.

### Rationing of care and services

CCWs were continuously re-evaluating the conditions and needs of their clients. As a result, care was tailored to the needs and circumstances of each client, whether this was explicit or not. The ranking of clients and their needs by priority was not a one-off process, but CCWs were repeatedly revising decisions about which clients were seen first, what form of support was provided, and at what point in the care process.

#### Tailoring care services

The process of rationing care and services resulted in the repeated tailoring of services for each client. Tailoring of services began prior to the CCW's arrival at the household, particularly in the context of clients who were already enrolled and known. The rationing and tailoring processes not only affected the type of care provided, but also the frequency with which care was provided. Therefore, the daily schedule of visits to clients was tailored according to some form of ranking or prioritisation assigned accordingly by the CCW. In the quote below, a CCW explains how she prioritised clients she would visit during her work day.

Each day I see seven clients and it depends on the conditions of the clients, because they are staying far from each other, and I have to see those who have serious problems on that day. I know my clients, so then I have to start with those who need me to bathe them and to give them treatment; I will lastly see those who have minor problems. (CCW 17)

On the whole, the severity of the medical or physical condition took precedent over other needs, and such clients were seen first and/or more frequently by the CCW.

#### Value judgements in care

While severity of illness could be assessed through clinical grading or triage, CCWs tended to conduct a clinical assessment of their clients intuitively using their general knowledge, training, and experience, which might at times be limited. Therefore, the value of judgements placed by CCWs on the significance of their observations likely influenced their assessments and rationing of care services provided.

Mrs. M. is suffering from a stroke of her entire body. She is not able to turn, and she is bedridden. I have to feed her, turn her on her side, and massage the side on which she was sleeping. She also has a wound in her leg and she is not able to see it. She just heard that she has a wound. I used to wash that wound, apply ointment, and dress it and lay her where she was sleeping. (CCW 3 about Client 6, stroke)

The logic of rationing care was not only restricted to CCWs, but could also be seen in the way PCGs understood the allocation of roles. In the example below, the PCG regarded cooking, feeding, and treatment as critical parts of care, and the presence of the CCW was not for domestic relief, but to care for the patient. Therefore, services that were seen to be outside of that domain were for the family to handle.

She [CCW 13] cooks food for patient and asks about her pills. She is helping the patient. We would never ask her to do anything like sweep the yard. She is here to help the patient only. She could even bathe her. But you find that we have already done it. We do this in the morning and in the afternoon. (PCG 28 about Client 28, female, stroke)

#### Intuitive subjective needs assessment

While in one household sweeping of the yard by the CCW was not acceptable to the family, in another household the same service was necessary, particularly for clients living in solitude. In the following quote, the repeated use of the word ‘if’ brought to light or perhaps confirmed the unstructured manner in which the service package was rationed for each household and that decision-making was a joint negotiated process.

I would bathe patients and sweep the yard if I found them living alone. If the patient is in a bad condition, I know I would go clean the house, wash the dishes, make a fire, and cook for them and also wash their clothes if they are dirty. If they had an iron, I would iron their clothes. They have to be clean like I am. I had to look at myself to see how I was and what I was wearing. That is what I also had to do for the patient. (CCW 3)

The use of ‘self’ as a reference point indicated the subjectivity in the way rural HBC was rationed and decided upon, highlighting the lack of objective standardisation. However, the ‘self’ reference could simply be a representation of the narrow sociocultural distance between the CCW and the client, revealing a lens that allows CCWs to see aspects of their own selves in their clients. In either case, it remained evident that some CCWs were extending themselves through their worldview to provide the best possible care for the client, as they would have done unto themselves.

### Appraisal of care and services

There were consequences and outcomes of needs assessment and rationing of care carried out by CCWs, in that clients subjected them to scrutiny and criticism.

#### Critique of tailored care services

Some clients were not aware of the decision-making and rationing process followed by the CCWs when care was not explicitly negotiated, although an understanding could be established intuitively. Consequently, some clients deemed the rationing process inequitable, unjust, and unfair.

There is a young lady [CCW] who comes to check on me but she does nothing. I mean in other places they clean the room and with those with no one to cook for them the CCW cook for them. (…). They do nothing for us though it may be because I am still young. But for grandmother [the grandmother of the client who passed away used to receive HBC] they used to bathe her and the lady would go into granny's room and clean too. (…). I hear people say that if you are living alone and sick they even clean your house but for me I think it is because I have children who can take care of me. (Client 18, female, diabetic)

The above client was protesting against what she considered to be unequal treatment, using other people as benchmarks, while unaware of the needs assessment and tailoring processes followed by the CCW. However, the client was able to note that having children who cared for her may have been associated with the differential treatment she received from the CCW. CCWs confirmed that their rationing of care took into account the help already available to their clients, including those from clients’ children, as shown below.

I don't really do anything. Her daughter does all the cooking and cleaning for her. I just go there to check up on her and to remind them not to get tired of always taking the treatment and going to the clinic. (CCW 13 about Client 29, female, heartburn, mobility impairment)

#### Monitoring of needs and tailoring of care

CCWs continued to monitor the care of their clients, even when clients did not seem to appreciate support services that were not tangible. One of the clients (Client 12, female, HIV/AIDS) explained, ‘When they [CCW] are here they do ask my child who looks after me if I ate or took a bath, and they also check my bedroom area to see if it's clean. They always do any of these above-mentioned things if my daughter has not done them’. The PCG confirmed, ‘She [CCW 5] does not have any particular tasks’ (PCG 12).

#### Appreciation of tailored care services

In cases where CCWs were not allowed to provide tangible services, support visits appeared to be welcome and were found to be sufficient.

When I go to greet them, she [PCG 29] does tell me that my coming is enough because Client 29 will never allow me to touch any of her things, whether it's her laundry or anything. She only wants her daughter to touch her stuff. (CCW 13 about Client 29, female, heartburn, mobility impairment)

For such households, there was much enthusiasm arising from the perception and recognition of the difference made by the presence and work of the CCWs.

### Reinforcing the social support system

The care provided to the client was often a shared task between the CCW and other members of the social support network, including the PCG. Since the care burden for a CCW was fairly high due to large numbers of clients they cared for, mobilising social support systems for clients was a crucial step. Clients and members of their social support systems also had preference on how care tasks should be shared.

#### Division of labour in functional social support networks

In this study, the social support system comprised of family members, nuclear and extended, and neighbours or friends. Children often formed the most important part of the social network structure available to HBC clients. Within the social support network, there was division of labour, whereby different members of the support system each played a part in caring for the client, as demonstrated below.

My wife used to bathe me after work, my mother gave me food during the day, and my sister was the one taking me to hospital. (…) Those were the tasks of my family in my life. (Client 13, male, HIV/AIDS)

#### Supporting weakened social support networks

When clients had functional support systems, the amount of work done by the CCW was reduced because the workload was shared among several people. Sometimes the existing social support system was unable to provide all or part of the essential care needed, or the family caregivers were not able to carry out certain tasks, in which case the CCW had to provide extra support to the household.

Ahhh … last year she [PCG 8] was involved in a car accident and her hand got broken. She could not even sit for her final matric exams. Her fingers from that time never healed properly so she struggles with everything she does. She has tried getting help but doctors are saying that there is nothing that can be done. In other words she cannot fetch water; it is only mama K [CCW 4] who helps with that. (Client 8, female, TB and high blood pressure)

#### Identifying failing social support networks

In certain circumstances, the support network was small, and the PCGs were overwhelmed with care work, even when they were motivated by a sense of duty to assist the client, as the PCG below described.

I am doing this all alone. If I had someone helping me then it would be better because they would do this while I bathe my mother. It's easier when you have a helper. I am not saying it's hard on me in a bad way. (…). I feel that it is my duty to take care of my parent because when I also have a problem, when she is well enough, she is able to help me. (PCG 28 about Client 28, female, stroke)

The CCW, given their intuitive needs assessment and flexible approach to negotiated care, were able to offer respite to PCGs.

She [CCW] helps with fetching water and believe me, if it was not for her, we do not know how things would be in this house. (PCG of Client 8, high blood pressure)

#### Expanding the social support network

When the social networks of the clients were inadequate and the CCWs were not able to provide all the necessary care, CCWs made efforts to expand such social networks. CCWs would either mobilise family members to get involved in the care process or, when these were not available, alternate people including neighbours or other community members were approached with the client's permission and requested to assist with the care provision.

When we [CCWs] go to a patient and find that he is unable to bathe himself, we do it for him. When he is unable to cook we cook for him. We visit him regularly when he lives by himself. Or we simply ask him which of his neighbours he gets along very well with. We sit down with the neighbour and ask her to be our eyes when they get off work at night and on weekends. The neighbour would then give us a report on how everything was with the patient. (CCW 15)

## Discussion

In this study, by investigating the process of approaching delivery of HBC by CCWs within a rural setting, we found several lessons that have implications for the general understanding of care management and the quality of the services delivered by CCWs in the context of social support networks. First, CCWs conduct an intuitive assessment of needs for the client, the PCG, and the household. Through a process of negotiated care with the client and the PCG, the CCW settles on a tailored client-centred package of care mindful of the client's personal boundaries of preference. Second, the CCWs engage in a cyclical process to evaluate and adapt the package of services for each client and their household whenever the client's circumstances change for better or worse. The PCGs are also engaged in the same process of rationing care and decision-making in terms of what needs to be done by whom, such that the burden of care is distributed and shared among these providers on a case-by-case basis. Third, the clients are involved in appraisal of care services provided by the CCWs or the PCG, and occasionally PCGs also actively appraise the CCWs, in which the recipients of care decide whether the package of services is acceptable or not. When the package of services is poorly negotiated or communicated, client dissatisfaction with care may be increased, resulting in strained relationships. Finally, CCWs tend to actively reinforce a social support system for the client, such that a stronger social network can develop around the client.

Currently, there are no known standardised methods for determining needs for health care in rural HBC, and if such tools were to be employed their suitability for client-centred care remain to be seen. With increasing calls for the professionalisation of CCWs (20, 21), there is a risk of over-standardisation in approaches to client care, which may arguably compromise the ability of CCWs to apply their intuitive assessment of needs for clients and their households. This study showed that service packages are, albeit crudely, uniquely crafted for each household, and the preferences of each client remain central to the process of negotiating care. Top-down policy processes and program designs may inadvertently impact negatively on these current grassroots approaches. Although no evidence of standardisation is available within the developing world context, developed countries offer some evidence. Research in Denmark shows that regulated HBC does lead to the provision of care based on the needs of the elderly with complex problems (22). Care providers in this study indicated that they felt trapped in a dilemma between following the wishes of the elderly client and carrying out the tasks ordered by the authorities to maintain an acceptable standard of living. Implementing standards set out by care institutions or the government does not necessarily provide caregivers with the time, support, autonomy, or flexibility to provide care according to the needs of the client (22). Although there is a risk in standardisation of care, guidelines towards care provision might improve quality of care and a fair distribution of various types of care, including medical care, towards a standardised and acceptable benchmark.

Through a negotiation process led by the CCW, care provision often becomes a shared experience between the CCW, PCG, and the social support network. The importance of the availability of a social support network in HBC provision forms a gap in the current research. The literature focuses on the main caregiver and does not include the importance of the support caregivers receive from spouses and other nuclear family members, for example. Szinovacz and Davey conclude that more research is needed on how the support of social support networks influence care provision and the effect of the availability of these networks on clients, carers, and other family members (23). Although the availability of social support networks might offer relief in the burden of care often carried by the CCW, the vulnerability of these social support networks cannot be overlooked. Seeley et al. already highlighted the vulnerability of social support networks, as assumptions that the extended family will be able and willing to assist sick family members is not always the reality (24). The assumed ‘safety net’ of family for the client might become complex, with limitations and constraints, as family members fear stigma associated with a family member who is living with HIV or has died a ‘shameful death’ because of AIDS (25). Due to HIV/AIDS-related stigma, PCGs and CCWs might be closed off from social support when they need it (5), thereby increasing the burden of care. Although the role of stigma in health-seeking behaviour is not new, it can prevent carers from reaching out for support even when it is available in the community (26–28). A study by Dawson shows that in a household compounded by HIV/AIDS, related stigma undermines kinship ties and renders relationships with the extended family more conditional, temporary, and at times destructive (29).

The involvement of a PCG or other care provider is essential, especially when a client takes treatment. It is easier for PCGs than a CCW to be present on a day-to-day basis at the specific time that medications need to be taken. PCGs are therefore ideally positioned to augment treatment adherence over long periods of time. A study in Cambodia and Thailand by Knodel et al. concluded that family members commonly help with the ART treatment regimens of the client, including reminding them to take medication, particularly when they live in the same household (30). Family members can also assist in reminding the client to attend clinic appointments for collection of medication and may accompany them, a task often seen as complicated for a CCW (30). It is therefore not only in the best interest of the CCWs, but also the client, to ensure that these clients are well supported by their social support system. These benefits may explain why CCWs go to such great lengths to ensure that social support networks are created when absent or strengthened when weak. However, the literature on programs that support CCWs still needs to identify the creation and strengthening of social networks and support systems as an essential skill set for CCWs in resource-poor settings. Currently, these processes are carried out through intuitive and unstructured subjective methods, which may also be the most suitable, since CCWs tend to know and understand the context of their clients.

We acknowledge that the impact of the work done by CCWs, including the quality of care provided, should also be measured in terms of the morbidity and mortality of their clients (31, 32). However, we also believe that outcome measures should relate directly to inputs and processes, while taking into account the roles played by other members of the social support networks for HBC clients. Limitations in this study include its qualitative nature, which constrain the generalisability and transferability of the findings, and may have been affected by social desirability bias. However, triangulation of data collection and a theory-driven approach to this study served to reduce the effect of limitations in study design. Furthermore, the novelty of these results will serve to close a crucial knowledge gap in our understanding of service delivery by CCWs. Understanding how care decisions are made will help us understand the challenges faced by care providers, both CCWs and PCGs, including an increased risk of burnout, vulnerability to illness, and emotional despair (33). The implications for the PCG's and CCW's personal health and well-being can be enormous (33), resulting in carers becoming exhausted and running out of options to fight the challenges they face, a potential devastation for all involved. With an improved understanding of how tasks are divided, tailored, and optimised between the family and the CCW, better risk protection strategies can be developed and implemented.

## Conclusions

In this study, CCWs allowed clients to guide and determine care services to be delivered, and these varied between clients. Furthermore, clients showed increased acceptability and satisfaction with care when their desires were honoured by their care team and family members. Therefore, while the current approaches to HBC in rural and low resource settings may not be of high technical design, they appear to align with the notions of patient-centred care. The dimensions of care include the role of the PCG and the client, as well as the social support network available and the strength of the network ties that bind them. The study suggests that HBC provision in rural South Africa is largely intuitive, and involves a needs assessment, negotiation of services, joint decision-making, and rationing of care support. Sharing the burden of care through the establishment or strengthening of a social support system is essential and guides the rationing of care by the CCWs. Therefore, evaluators of quality in HBC delivered by CCWs in rural settings should ask the following questions: 1) What are the needs of the client and gaps in care and support? 2) Who is available in the social support network to provide what kind of care or support to the clients? 3) Was the care or support well received by the client? In this way, the quality of care measures can be adapted to fit the approach to care implemented by CCWs.

## Paper context

Community Care Workers (CCWs) form a crucial part in providing home-based care (HBC). However, the largest share of care occurs within their clients’ social support networks, the dynamics of which were poorly understood. The study indicates that, through CCWs, service packages are needs-based, constantly negotiated, cyclically monitored, uniquely crafted and tailored to clients, and aligned to available social support networks. Implementers of HBC should consider assessment of clients’ needs, preferences, and social support networks, and tailored care.

## Authors' information

MM (MBChB, MFamMed, Dip HIV, PhD) is a health systems and policy researcher, with experience and interest in diverse resource-poor settings in sub-Saharan Africa. IS (MSc and PhD candidate) has an interest in the community-level quality of health care. FB (PhD) has extensive experience in Latin America and conducts health policy research.
